# Case report: A case of fulminant type 1 diabetes mellitus after COVID-19 vaccination during treatment of advanced gastric cancer: pitfall in managing immune-related adverse events

**DOI:** 10.3389/fonc.2023.1264281

**Published:** 2023-12-20

**Authors:** Toshimitsu Tanaka, Sachiko Nagasu, Takuya Furuta, Mizuki Gobaru, Hiroyuki Suzuki, Yasutaka Shimotsuura, Jun Akiba, Masatoshi Nomura, Fumihiko Fujita, Takumi Kawaguchi, Keisuke Miwa

**Affiliations:** ^1^Multidisciplinary Treatment Cancer Center, Kurume University Hospital, Kurume, Japan; ^2^Division of Gastroenterology, Department of Medicine, Kurume University School of Medicine, Kurume, Japan; ^3^Department of Surgery, Kurume University School of Medicine, Kurume, Japan; ^4^Department of Diagnostic Pathology, Kurume University Hospital, Kurume, Japan; ^5^Division of Endocrinology and Metabolism, Kurume University School of Medicine, Kurume, Japan

**Keywords:** Fulminant type 1 diabetes mellitus, gastric cancer, immune-related adverse event, nivolumab, COVID-19 vaccination, Autopsy

## Abstract

The occurrence of fulminant type 1 diabetes mellitus as an adverse event during cancer immunotherapy has been previously reported. However, little is known about the causal relationship between the coronavirus disease 2019 (COVID-19) vaccination and fulminant type 1 diabetes mellitus. A 60-year-old man with advanced gastric cancer, receiving S-1 + oxaliplatin and nivolumab therapy, followed by nab-paclitaxel + ramucirumab as a second-line treatment, with steroid supplementation for complications of hypopituitarism-induced hypoadrenocorticism, was administered a COVID-19 vaccine after three cycles of nab-paclitaxel + ramucirumab. Two days later, he developed severe malaise and anorexia, which required emergency admission to our hospital for suspected adrenal insufficiency. Despite increasing steroids, his general condition changed suddenly after 12 hours leading to his death. Histopathological analysis of autopsy samples revealed loss of the islets of Langerhans, indicating fulminant type 1 diabetes mellitus. We failed to recognize the onset of fulminant type 1 diabetes mellitus because its symptoms were similar to those of adrenal insufficiency. The number of reports on the onset of fulminant type 1 diabetes mellitus after COVID-19 vaccination has been increasing, and in this case, the onset occurred on the second day after COVID-19 vaccination, suggesting an association between vaccination and fulminant type 1 diabetes mellitus. Clinicians should be aware of the risk of fulminant type 1 diabetes mellitus, although rare, after COVID-19 vaccination.

## 1 Introduction

In treatment for advanced gastric cancer, recently, the approval of combination therapy involving, nivolumab, an immune checkpoint inhibitor (ICI) that blocks programmed cell death-1, and chemotherapy as first-line treatment has resulted in the increased overall survival and progression-free survival of patients (1, 2). ICIs, such as nivolumab, can cause imbalances in the immunological tolerance of patients and inflammatory side effects known as immune-related adverse events (irAEs), including pneumonitis, colitis, hepatitis, pancreatitis, and hypophysitis (3). While appropriate management can ameliorate most irAEs, severe irAEs can be fatal (4).

The COVID-19 pandemic is now a global public health problem; as the COVID-19 pandemic continues, COVID-19 vaccine development has progressed rapidly and several COVID-19 vaccines are now approved worldwide (5). Many cancer patients and healthy people have been vaccinated. In recent years, there have been increasing reports of patients developing fulminant type 1 diabetes mellitus (DM) after receiving the COVID-19 vaccine (6–12). However, no report has yet clarified the pathological findings of fulminant type 1 DM.

Here, we report the case of a patient who received the COVID-19 vaccine during steroid replacement therapy for hypopituitarism induced by immunotherapy for advanced gastric cancer and subsequently developed fulminant type 1 DM, resulting in death.

## 2 Case description

A 60-year-old Japanese man with unresectable advanced gastric cancer with multiple liver metastases (TNM cT3N3M1, stage IVB, well-differentiated adenocarcinoma) presented to our hospital in December 202X. The patient was initially treated with the SOX (S-1 + oxaliplatin) + nivolumab regimen (consisting of oral S-1 40 mg/m^2^ twice daily on days 1–14, intravenous oxaliplatin 130 mg/m² on day 1, and nivolumab 360 mg/body every 3 weeks) as first-line chemotherapy + immunotherapy. However, during the first cycle, the patient experienced grade 3 (CTCAE, version 5.0) diarrhea as an adverse event related to S-1; therefore, the chemotherapy regimen was changed to CAPOX (consisting of oral capecitabine 1000 mg/m² twice daily on days 1–14 and intravenous oxaliplatin 130 mg/m² on day 1, every 3 weeks). However, 4 months later, computed tomography revealed progressive disease due to primary tumor growth. The chemotherapy regimen was thus switched to nab-paclitaxel (100 mg/m² intravenously on days 1, 8, and 15) + ramucirumab (8 mg/kg intravenously on days 1 and 15) as second-line chemotherapy in April 202X+1. After the second cycle of treatment was initiated, anorexia, fatigue, and hyponatremia (116 mmol/L, reference interval: 138–145 mmol/L) were observed. His serum cortisol (0.36 μg/dL, reference interval: 6.24–18.00 μg/dL) and adrenocorticotropic hormone (ACTH) levels (<1.5 pg/mL, reference interval: 7.2–63.3 pg/mL) had decreased. The patient was referred to an endocrinology and metabolism specialist, who conducted a loading test and diagnosed him with hypopituitarism-induced adrenocortical hypofunction as an irAE at 5 months after the initial administration of nivolumab. Steroid therapy (hydrocortisone 20 mg/day) was immediately initiated, and his anorexia and fatigue improved. Consequently, steroid and nab-paclitaxel + ramucirumab therapy were continued. During the treatment course, the patient received the third dose of the COVID-19 vaccine (Pfizer-BioNTech, NY, USA). However, on the second day after vaccination, he suddenly developed severe anorexia and fatigue, requiring urgent admission to our hospital in June 202X+1 (6 months after the initial administration of nivolumab). The laboratory and genetic data obtained upon admission are detailed in **Table 1**.

**Table 1:** Laboratory data upon admission.

Biochemistry		Peripheral blood	
Total protein	7.4 g/dL	White blood cell	4,400/μL
Albumin	4.2 g/dL	Neutrophil	55.7%
Total bilirubin	0.9 mg/dL	Lymphocyte	28.7%
Aspartate aminotransferase	15 IU/L	Monocyte	9.5%
Alanine aminotransferase	16 IU/L	Eosinophil	5.4%
Lactate dehydrogenase	183 IU/L	Basophil	0.7%
Alkaline phosphatase	123 IU/L	Red blood cell	419×10^4^/μL
γ-glutamyl transpeptidase	68 IU/L	Hemoglobin	11.5 g/dL
Amylase	59 IU/L	Hematocrit	37.4%
Blood urea nitrogen	23 mg/dL	Platelet count	17.7×10^4^/μL
Creatinine	0.84 mg/dL	**Endocrine hormone**	
Uric acid	8.9 mg/L	TSH	2.35 mIU/L
Serum sodium	130 mEq/L	Free T3	1.6 pg/mL
Serum potassium	4.8 mEq/L	Free T4	1.38 ng/dL
Serum chloride	89 mEq/L	ACTH	<1.5 pg/mL
C-reactive protein	7.16 mg/dL	Cortisol	8.16 ug/dL

TSH, thyroid stimulating hormone; T, triiodothyronine; T4, thyroxine; ACTH, adrenocorticotropic hormone.

**Figure 1:**
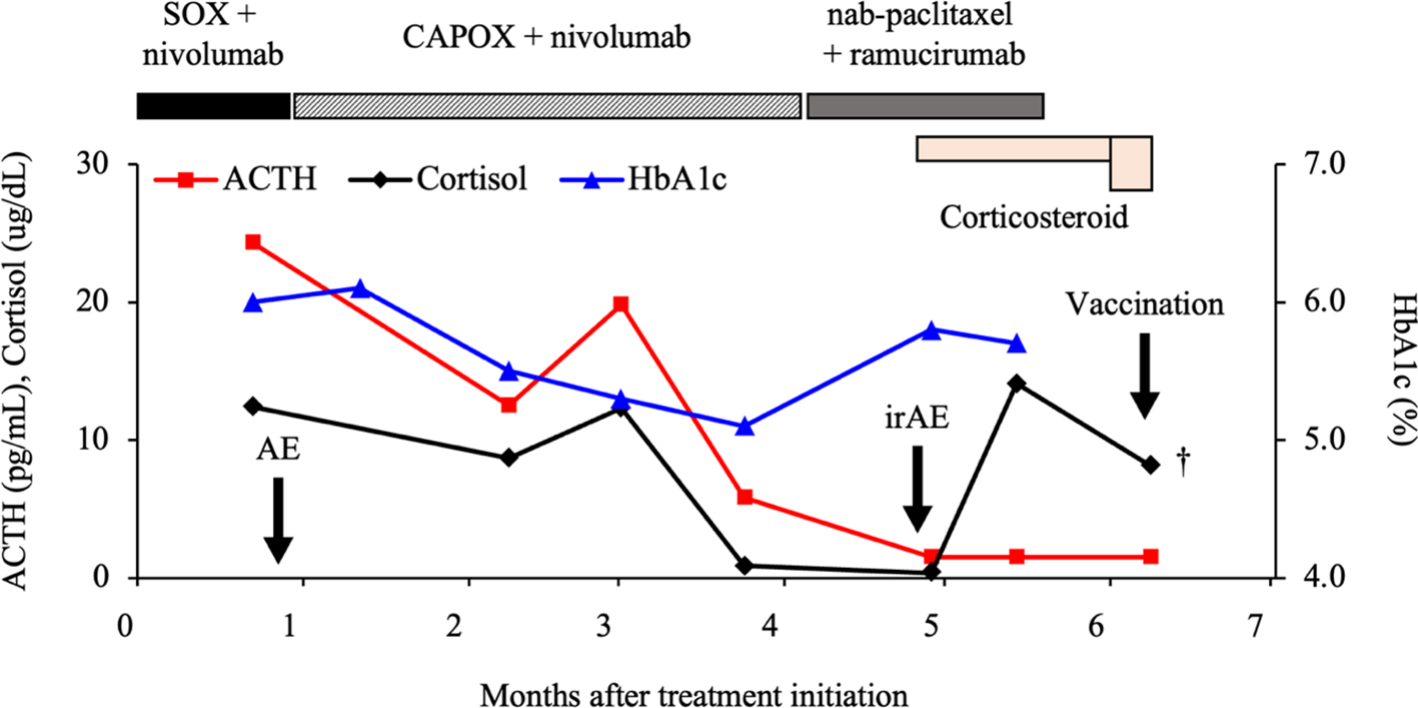
Clinical course of the patient. ACTH, adrenocorticotropic hormone; SOX, S-1 + oxaliplatin; CAPOX, capecitabine + oxaliplatin; HbA1c, hemoglobin A1c; AE, adverse event; irAE, immune-related AE.

The symptoms of appetite loss and fatigue and laboratory findings indicating hyponatremia meant that he was in a state of adrenal insufficiency. We, therefore, increased the hydrocortisone dose to 50 mg/day and initiated infusions to correct the hyponatremia. However, the patient’s symptoms did not improve and his fatigue worsened. Twelve hours after starting the treatment, sudden decline in his consciousness level and blood pressure was observed. His blood glucose level at this time was markedly elevated, exceeding 1,000 mg/dL. Insulin was immediately administered, but he soon experienced cardiopulmonary arrest. Despite attempting cardiopulmonary resuscitation, his heartbeat did not resume and he died (**Figure 1**). With the family’s consent, an autopsy was performed to determine the cause of death.

**Figure 2:**
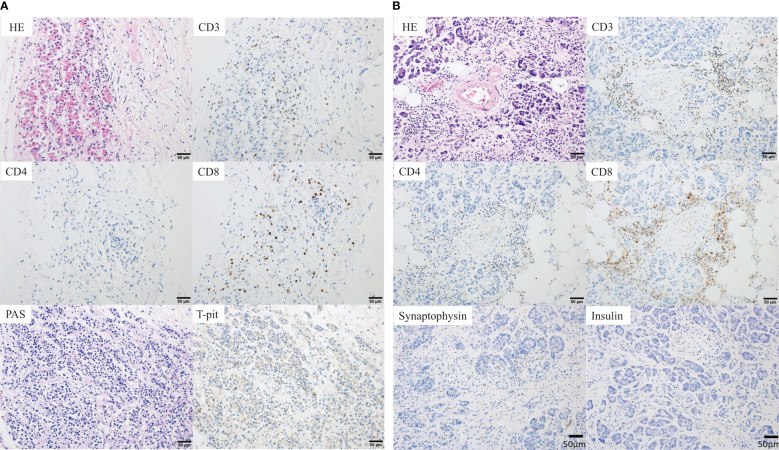
Pathological findings at autopsy. **(A)** Anterior pituitary gland. Histopathological findings showing focal lymphocytic infiltration comprising CD3^+^ T cells, suggesting cellular damage to the anterior pituitary gland. The number of infiltrating cells was 1, 0 and 1 per three high power fields (HPFs) for CD4^+^ T-cells and 62, 44, and 57 three HPFs for CD8^+^ T-cells. In addition, the negative periodic acid-Schiff (PAS) staining and negative T-Pit expression suggested that the number of ACTH-producing cells was reduced. **(B)** Pancreas. The pancreas showing marked interstitial fibrosis and moderate CD3^+^ T-cell infiltration (CD4<CD8), suggesting cellular damage to the pancreas. The number of infiltrating cells was 28, 14, and 13 per three HPFs for CD4^+^ T-cells and 107, 52 and 71 per three HPFs for CD8^+^ T-cells. Moreover, there were no synaptophysin- and insulin-positive cells, and the islets of Langerhans were completely absent. ACTH, adrenocorticotropic hormone; HE, hematoxylin and eosin staining.

Histopathological examination of the anterior pituitary gland revealed the presence of a focal lymphocytic infiltrate comprising CD3^+^ T cells (CD4<CD8). Negative periodic acid-Schiff staining and negative T-Pit expression indicated a decrease in ACTH-producing cells (13). Hence, the diagnosis of hypopituitarism due to selective disruption of ACTH-producing cells, an irAE induced by nivolumab, was established (**Figure 2A**). However, the absence of severe inflammation and necrosis indicated that it was not the direct cause of death. The structure of the adrenal cortex was intact, no inflammatory cells were observed, and mild lymphocytic infiltration was observed in the adrenal medulla; these were considered physiological occurrences and not the cause of death. The pancreas exhibited marked interstitial fibrosis, moderate CD3^+^ T-cell infiltration, with a similar pattern to that of the pituitary gland (CD4<CD8), and absence of synaptophysin- and insulin-positive cells and the islets of Langerhans (**Figure 2B**). More than two-thirds of the cancer cells in the primary gastric lesion were pathologically necrotic, and the liver metastases were completely necrotic. Therefore, the patient was diagnosed with fulminant type 1 DM. Considering the histopathological findings and clinical course, we concluded that the patient developed severe diabetic acidosis due to rapid hyperglycemia that led to circulatory failure and death.

## 3 Discussion

Nivolumab + chemotherapy has become the standard first-line treatment for advanced gastric cancer after improved outcomes were observed in the CheckMate 649 and ATTRACTION-4 trials (1, 2). However, ICIs such as nivolumab can cause immune activation in non-target tissues, leading to irAEs in some patients (3). In the ATTRACTION-4 study, irAEs involving endocrine disturbance were reported in 11.4% of the patients, with 2.2% experiencing irAEs of grade 3–4 severity (2). Pituitary insufficiency, often presenting as ACTH deficiency, occurs in approximately 6% of the patients on ICIs (14, 15). The most severe form of pituitary insufficiency is ACTH insufficiency; it can lead to acute adrenal insufficiency and be fatal if treatment is delayed. Further, the prevalence of type 1 DM as an endocrine-related irAE due to nivolumab is <0.2% (16). Therefore, type 1 DM is considered a rare irAE. Although there are several reports of hypopituitarism and DM as irAEs caused by ICI (17, 18), to our knowledge, there is no report of hypopituitarism-induced adrenal hypofunction combined with fulminant type 1 DM.

Fulminant type 1 DM, a subtype of type 1 DM, was first identified in Japan and is commonly diagnosed in Asian populations (19). It is characterized by severe hyperglycemia with ketosis, nearly normal hemoglobin A1c (HbA1c) level, and lack of insulin secretion at onset. Other features include absence of detectable levels of islet autoantibodies, elevated levels of pancreatic enzymes, and frequent influenza-like symptoms before DM onset (14). In our patient’s case, although the HbA1c level was within the normal range, since the tests for acidosis and insulin secretory capacity were not performed and the diagnostic criteria for fulminant type 1 DM (16) were not met, hyperglycemia was observed just before death. Pathological findings from the autopsy revealed the near-complete disappearance of the islets of Langerhans, leading to the diagnosis of fulminant type 1 DM. In addition, no insulin antibodies were present in the islets of Langerhans, suggesting endogenous insulin deficiency. The presence of CD3^+^ T cells and predominance of CD8^+^ cells in the pancreatic tissue suggest that an immune response contributed to the destruction of pancreatic beta cells and development of fulminant type 1 DM. However, cases of fulminant type 1 DM after COVID-19 vaccination even without prior ICI treatment has been reported (**Table 2**), and these findings alone cannot distinguish between fulminant type 1 DM due to irAE and fulminant type 1 DM as a pure adverse event of COVID-19 vaccination. In the future, the histopathology of fulminant type 1 DM should be carefully monitored.

**Table 2:** Reports of fulminant type 1 DM after COVID-19 vaccination.

Author	Age (years), Sex	Vaccine	Times	Onset	Symptoms	History of DM	Prior ICI
**Kobayashi T** (6)	59, M	mRNA	2	15 weeks	weakness, fatigue, difficulty in body movements	−	−
**Tang X** (7)	50, M	inactivated	1	6 days	polydipsia, polyuria	−	−
**Nishino K** (8)	77, F	mRNA	2	2 days	disturbance of consciousness	+	+
**Ohuchi K** (9)	45, M	mRNA	2	3 days	fatigue, dry mouth, weight loss, increased urine frequency	−	+
**Sasaki K** (10)	45, F	mRNA	1	3 days	fatigue, thirst, nausea, abdominal pain	−	−
**Huang L** (11)	39, F	protein subunit	4	6 days	fatigue, thirst, nausea, vomiting	−	−
**Sakurai K** (12)	36, F	mRNA	1	3 days	polydipsia, polyuria, palpitations, loss of appetite, fatigue	−	−

DM, diabetes mellitus; COVID-19, coronavirus disease 2019; M, male; F, female; ICI, immune checkpoint inhibitor. "-" indicates "No" and "+" indicates "Yes".

The initial irAE was adrenal insufficiency due to hypopituitarism, which showed improvement with hydrocortisone administration. The patient’s chief complaint was anorexia and fatigue, and blood tests revealed decreased cortisol, ACTH, and sodium levels, supporting the diagnosis of severe adrenal insufficiency. In addition, we did not suspect type 1 DM, which is an infrequent complication, as there were no findings suggestive of metabolic ketoacidosis, such as Kussmaul breathing or acetone odor. However, pathological findings from the autopsy revealed no adrenal insufficiency, and hypopituitarism caused by irAEs was mild and not a direct cause of death. Consequently, the increased hydrocortisone dose may have aggravated the type 1 DM, leading to death.

As shown in **Table 2**, an increasing number of reports has recently suggested an association between COVID-19 vaccination and fulminant type 1 DM (6–12). Hence, the disease may develop relatively early and with a variety of symptoms, regardless of the type of vaccine, number of vaccinations, history of DM, and prior treatment. The vaccine may have triggered the disease in this case as well. Although recent reports suggest that the COVID-19 mRNA vaccine sometimes exacerbates autoimmune diseases (15), the mechanism by which the COVID-19 vaccination causes fulminant type 1 DM remains unclear.

Our patient who received the COVID-19 vaccine during steroid replacement therapy for advanced gastric cancer subsequently developed fulminant type 1 DM. Had we been aware of the possibility of fulminant type 1 DM, we could have detected it early and saved the patient. Clinicians should keep in mind the possibility of their patient developing fulminant type 1 DM, although rare, after COVID-19 vaccination.

## Data availability statement

The raw data supporting the conclusions of this article will be made available by the authors, without undue reservation.

## Ethics statement

Ethical approval was not required for the studies involving humans because, since this is a case report of a specific subject for publication in a journal, our hospital does not require Ethics Committee approval, provided that personal information is kept in mind and written informed consent is obtained from the subject or the subject’s family. The studies were conducted in accordance with the local legislation and institutional requirements. The participants provided their written informed consent to participate in this study. Written informed consent was obtained from the patient's next of kin for the publication of this case report.

## Author contributions

TT: Writing – original draft. SN: Data curation, Writing – review & editing. TF: Investigation, Writing – review & editing. MG: Investigation, Writing – review & editing. HS: Visualization, Writing – review & editing. YS: Data curation, Writing – review & editing. JA: Investigation, Writing – review & editing. MN: Investigation, Writing – review & editing. FF: Supervision, Writing – review & editing. TK: Supervision, Writing – review & editing. KM: Writing – original draft, Writing – review & editing.
